# Bioactive Secondary Metabolites from *Trichoderma* spp. against Phytopathogenic Fungi

**DOI:** 10.3390/microorganisms8060817

**Published:** 2020-05-29

**Authors:** Raja Asad Ali Khan, Saba Najeeb, Shaukat Hussain, Bingyan Xie, Yan Li

**Affiliations:** 1 Institute of Vegetables and Flowers (Plant Pathology Lab), Chinese Academy of Agricultural Sciences, Beijing 100081, China; asadraja@aup.edu.pk (R.A.A.K.); sabanajeeb831@gmail.com (S.N.); 2 Department of Plant Pathology, The University of Agriculture Peshawar, Peshawar 25130, Pakistan; shussa@aup.edu.pk

**Keywords:** secondary metabolites, phytopathogenic fungi, antifungal, *Trichoderma*, biological control

## Abstract

Phytopathogenic fungi, causing significant economic and production losses, are becoming a serious threat to global food security. Due to an increase in fungal resistance and the hazardous effects of chemical fungicides to human and environmental health, scientists are now engaged to explore alternate non-chemical and ecofriendly management strategies. The use of biocontrol agents and their secondary metabolites (SMs) is one of the potential approaches used today. *Trichoderma* spp. are well known biocontrol agents used globally. Many *Trichoderma* species are the most prominent producers of SMs with antimicrobial activity against phytopathogenic fungi. Detailed information about these secondary metabolites, when grouped together, enhances the understanding of their efficient utilization and further exploration of new bioactive compounds for the management of plant pathogenic fungi. The current literature provides the information about SMs of *Trichoderma* spp. in a different context. In this review, we summarize and group different antifungal SMs of *Trichoderma* spp. against phytopathogenic fungi along with a comprehensive overview of some aspects related to their chemistry and biosynthesis. Moreover, a brief overview of the biosynthesis pathway, action mechanism, and different approaches for the analysis of SMs and the factors affecting the regulation of SMs in *Trichoderma* is also discussed.

## 1. Introduction 

Plant pathogens cause significant losses, which have obstructed efforts to increase agricultural production. In spite of remarkable achievements in the development of chemical pesticides, plant breeding technologies, and different cultural practices, as well as other management strategies for the control of plant pathogens, losses due to disease remain a limiting factor in agricultural production throughout the world, including many developed countries [1]. Among plant pathogens, phytopathogenic fungi are one of the main infectious agents in plants, causing significant economic and production losses. Throughout the history of agriculture, plant pathogenic fungi have been devastating threats and the most diverse group of economic and ecological threats [2].

Several management strategies have been utilized for the control of fungal plant pathogens, including the use of chemical fungicides, the breeding of disease resistance varieties, and several other cultural practices. The excessive and continuous use of chemical fungicides cause serious hazardous concerns related to human, animal, and environmental health. Breeding for disease resistance is a long-lasting process. Though resistance genes have been incorporated successfully in plants for disease management, breeding has to be a continuous process because the pathogens evolve rapidly, break the resistance, and plants become susceptible. In advanced agriculture, most of the fungal plant pathogens can be controlled by modern management practices but epidemics with huge yield losses still occur. Recently, wheat blast outbreaks (*Magnaporthe oryzae*) and soybean rust (*Phakopsora pachyrhizi*) in several Asian countries caused devastating yield losses [3]. There is a need to explore alternate management strategies. The use of biological control agents and their secondary metabolites is one of the potential approaches that is consumer and environmentally friendly. 

Secondary metabolites (SMs) from microorganisms may have an antifungal role against agriculturally important phytopathogenic fungi [4]. Among different microorganisms, the species of the genus *Trichoderma* are the most potent biocontrol agents in use today because they produce a diverse range of antimicrobial SMs [5,6]. *Trichoderma* species secrete a plethora of metabolites into their vicinity while having minimal nutritional needs. These metabolites can be utilized for agricultural, industrial, and medical benefits and hence are important to humans. Several *Trichoderma* spp. exhibit antifungal activities against phytopathogenic fungi [7], in which different groups of SMs, such as terpenes, pyrones, gliotoxin, gliovirin, and peptaibols may be involved [8]. Comprehensive information about these SMs regarding their antifungal role against phytopathogenic fungi, when grouped together, will enhance the understanding of their efficient utilization and further exploration of new antifungal bioactive metabolites for the management of plant pathogenic fungi. The current literature provides the information about SMs of *Trichoderma* spp. in a different context [9,10,11,12]. In this review article, we summarize and group different antifungal SMs of *Trichoderma* spp. against phytopathogenic fungi, along with a comprehensive overview of some aspects related to their chemistry and biosynthesis. In addition, a brief overview of different approaches for the analysis of SMs, the mechanism of action of SMs, the general biosynthesis pathway, and factors influencing SM regulation in *Trichoderma* is also discussed.

## 2. Antifungal SMs Produced by *Trichoderma* spp.

### 2.1. Epipolythiodioxopiperazines

**Figure 1:**
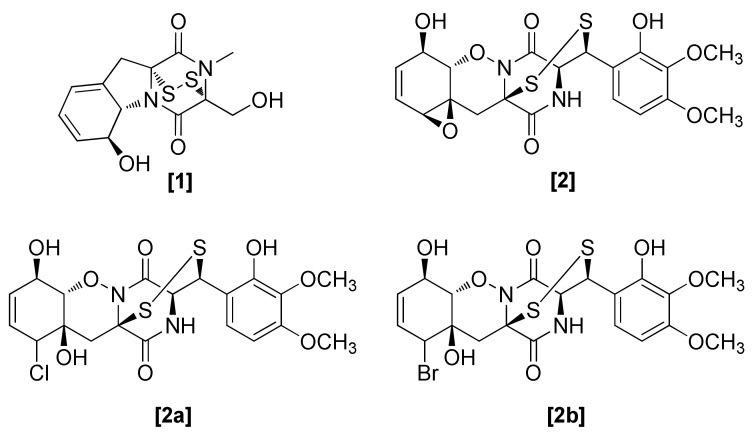
Structures of diketopiperazines from *Trichoderma* spp.: [1] gliotoxin isolated from *Trichoderma lignorum*, [2] gliovirin isolated from *T. virens*, [2a, 2b] analogues of gliovirin isolated from *T. longibrachiatum.*

**Figure 2:**
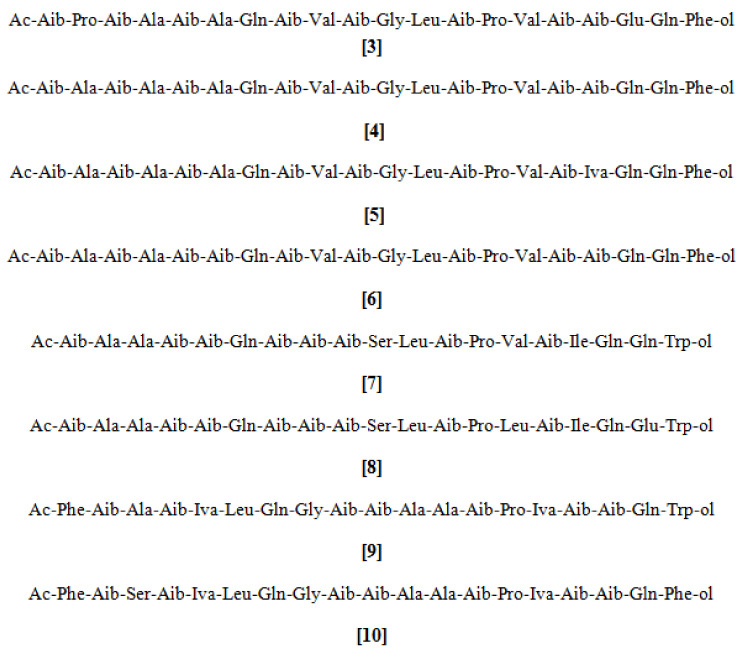
Structures of antifungal peptaibols from *Trichoderma* spp.: [3] alamethicin F30, [4] trichokonin VI, [5] trichokonin VII, [6] trichokonin VIII, [7] trichorzianine A1, [8] trichorzianine B1, [9] peptaivirin A, [10] peptaivirin B; peptaibols [4], [5], and [6] were isolated from *T. koningii*, [7], [8], [9], and [10] were isolated from *T. harzianum*.

Epipolythiodioxopiperazines (ETPs) have a high reactive potential among fungal SMs and are characterized by a diketopiperazine ring that originates from a peptide. Diketopiperazines (DKPs) are considered the product of protein degradation and they were generally nonpreferred peptides because they are synthesized from protein hydrolysates. The toxicity of ETPs is attributed to their disulphide bridges, which bind to thiol groups and generate reactive oxygen species through redox cycles, and in this way inactivate proteins [13]. In the past few years, scientists diverted more towards DKP research because of their strong biological activities. Many DKPs from microorganisms were isolated and studied for their biological activities. The first DPK gliotoxin (1) (Figure 1) was isolated from *Trichoderma lignorum* in 1936 [14], while a further description of gliotoxin was made from *Trichoderma viride* in 1944 [15]. Subsequent isolations and biosynthetic analyses have also been performed from this strain [16,17]. In 1975, Hussain et al. also isolated this compound from *Trichoderma hamatum*. Gliotoxins exhibit bioactivity against the human pathogenic fungus *Aspergillus fumigatus*, but also play important roles in the biocontrol activity of *Trichoderma virens* against some plant pathogenic fungi [18,19]. Some biocontrol strains (so-called Q-strains) of *T. virens* also produce gliotoxin [20]. For example, gliotoxin isolated from *T. virens* ITC-4777 was active against *Rhizoctonia bataticola* (with an ED_50_ of 0.03 g/mL), *Macrophomina phaseolina* (with an ED_50_ of 1.76 g/mL), *Pythium deharyanum* (with an ED_50_ of 29.38 g/mL), *Pythium aphanidermatum* (with an ED_50_ of 12.02 g/mL), *Sclerotium rolfsii* (with an ED_50_ of 2.11 g/mL), and *Rhizoctonia solani* (with an ED_50_ of 3.18 g/mL) [21]. Gliovirin (Figure 1; 2) is another member of this class of toxin, produces mainly by a strain of *T. virens* [22]. Two analogues of gliovirin (Figure 1; 2a, 2b) were isolated from *Trichoderma longibrachiatum*. These analogues exhibited antifungal activity against *R. solani* [23]. Strains of *T. virens* that produce gliotoxin also showed antagonistic activity against *R. solani* [24], while those strains that produce gliovirin were antagonistic to *Pythium ultimum* [25]. Both gliovirin and gliotoxin come under the epipolythiodioxopiperazine class of toxins and exhibited characteristic disulphide bridges [26]. The DKP gliotoxin gene cluster in the *T. virens* genome comprises eight genes, a cluster-specific regulator, auxiliary biosynthetic enzymes, and nonribosomal peptide synthetase (NRPS); dioxopiperazine synthetase [20]. The removal of a part from the *gliP* open reading frame confirmed the association of the gene cluster with gliotoxin production [27]. The gliP mutants that were unable to produce gliotoxin showed less activity against *P. ultimum* while exhibiting a higher vegetative growth rate [18]. Unexpectedly, another six genes of the *gli* cluster and *gliP* were also reported in the genome of *T. reesei*, but this species does not produce gliotoxin [20].

### 2.2. Peptaibols

**Figure 3:**
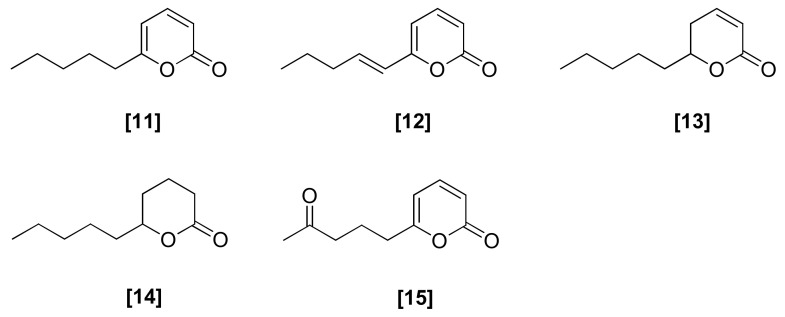
Structures of antifungal pyrones from *Trichoderma* spp.: [11] 6-PP isolated from *T. viride*; [12], [13], [14], and [15] analogues of 6-PP isolated from *T. harzianum*.

**Figure 4:**
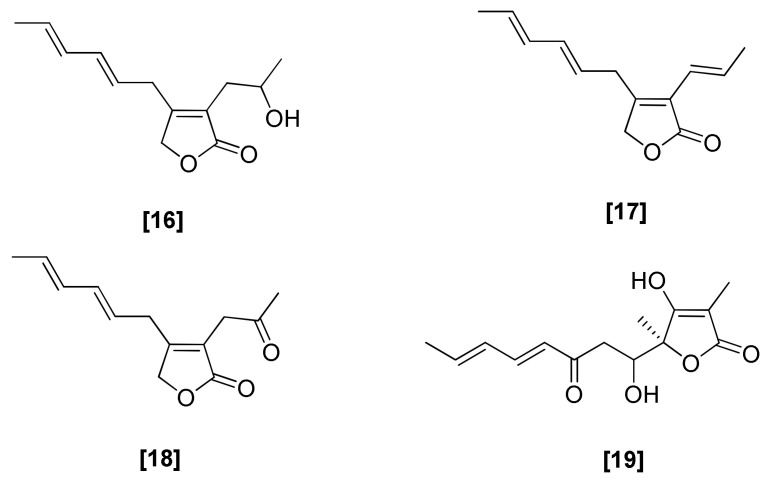
Structures of antifungal butenolides from *Trichoderma* spp. [16]: harzianolide, [17] dehydro-derivative of harzianolide, [18] T39butenolide, [19] 5-hydroxyvertinolide butenolides; [16], [17], and [18] were isolated from *T. harzianum* and [19] was isolated from *T. longibrachiatum*.

**Figure 5:**
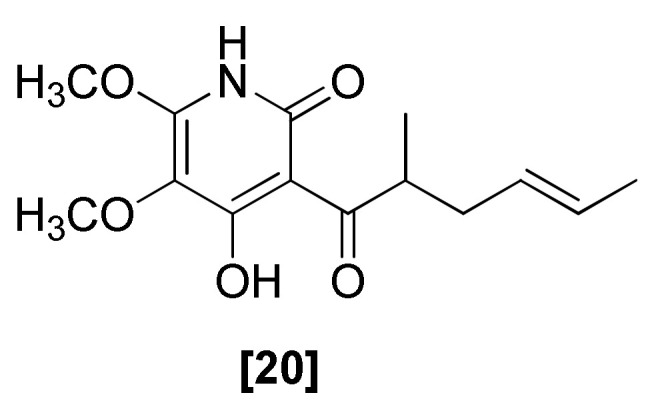
Structure of antifungal pyridone [20] harzianopyridone from *T. harzianum*.

**Figure 6:**
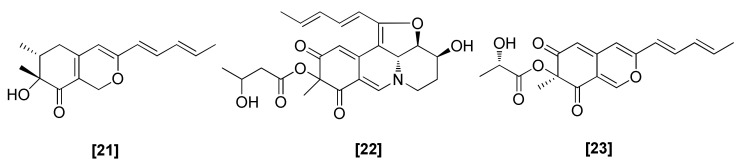
Structures of antifungal azaphilones isolated from *T. harzianum:* [21] harziphilone, [22] fleephilone, [23] T22azaphilone.

**Figure 7:**
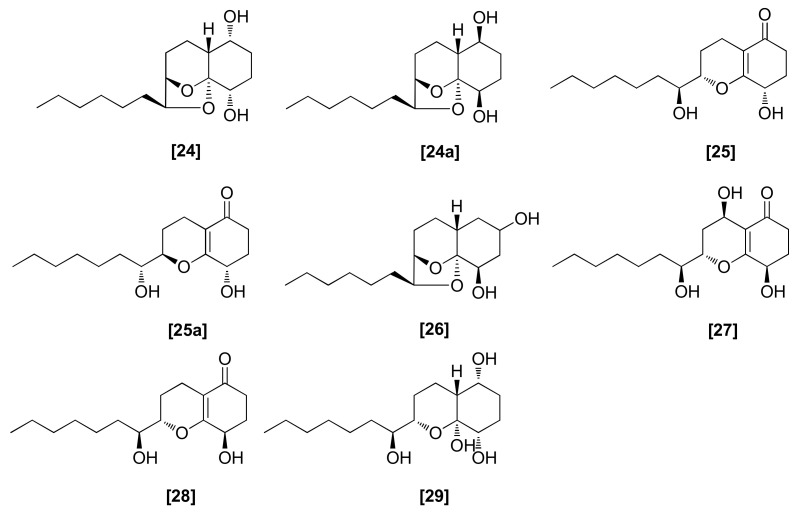
Structures of antifungal koninginins from *Trichoderma* spp.: [24] koninginin A, [25] koninginin B, [26] koninginin C, [27] koninginin D, [28] koninginin E, [29] koninginin G; koninginins [A], [B], [C], [D], and [E] were produced by *T. koningii* and koninginin [E] was produced by *T. aureoviride*.

**Figure 8:**
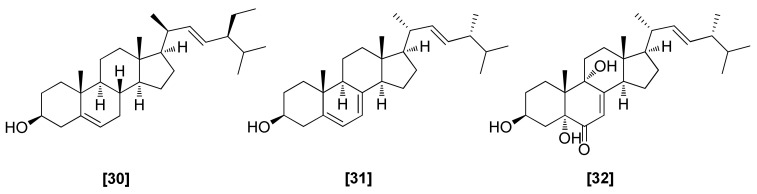
Structures of antifungal steroids from *Trichoderma* spp.: [30] stigmasterol, [31] ergosterol, [32] 3,5,9-trihydroxyergosta-7,22-dien-6-one.

**Figure 9:**
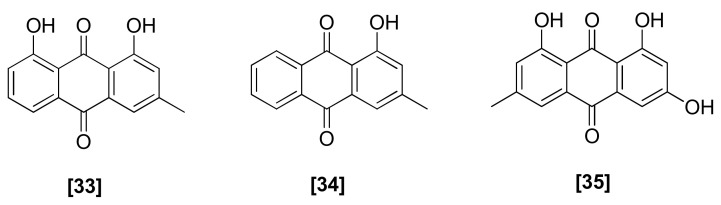
Structures of antifungal anthraquinones from *Trichoderma* spp.: [33] 1,8-dihydroxy-3-methylanthraquinone, [34] 1-hydroxy-3-methylanthraquinone, [35] 6-methyl-1,3,8-trihydroxyanthraquinone.

**Figure 10:**
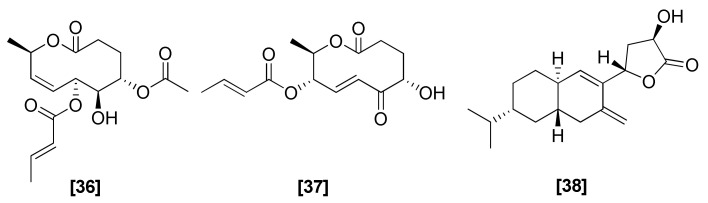
Structures of antifungal lactones from *Trichoderma* spp.: [36] cremenolide, [37] aspinolide C, [38] cerinolactone.

Peptaibols are the linear peptides consisting of α,α-dialkylated amino acids, isovaline, α-amino isobutyric acid (Aib), an acetylated *N*-terminus, and a *C*-terminal amino alcohol. They are ecologically and commercially important for their antimicrobial and anti-cancer properties, as well as their ability to induce systemic resistance in plants against microbial invasion. The peptaibols are amphipathic in nature and self-assemble to form voltage-dependent ion channels in membranes. This ability is largely responsible for the antibiotic properties of these compounds [28,29]. Peptaibols are produced largely by members of genus *Trichoderma* [30], and the first discovered peptaibol, alamethicin F30 (Figure 2; 3), was reported from *T. viride* [31,32]. Peptaibol subclasses were defined on the basis of peptide chain length. Those peptaibols having 18–20 residue peptides in their chain length are called long-sequence peptaibols [33,34,35,36,37], those having 11–16 residue peptides in their chain length are termed short-sequence peptaibols [38], while peptaibols having only 7–11 residue peptides in their chain length, with *N*-terminal amino acids acylated by a short lipid chain, are termed lipopeptaibols [39]. Three peptaibols, trichokonins VI (Figure 2; 4), VII (Figure 2; 5), and VIII (Figure 2; 6), obtained from *Trichoderma koningii*, showed broad-spectrum antimicrobial activity against a range of important plant pathogens, such as *R. solani*, *Fusarium oxysporum*, *Verticillium dahliae*, and *Botrytis cinerea*. Trichokonins are insensitive to proteolytic enzymes and showed biological activity over a wide pH range even after autoclaving [40]. Trichokonin VI (Figure 2; 4), isolated from *Trichoderma pseudokoningii*, induced extensive apoptotic programmed cell death in *Ascochyta citrullina*, *B. cinerea*, *F. oxysporum*, *Phytophthora parasitica*, and *V. dahliae* [41]. Interestingly, trichokonins were also proved to be highly active against *Clavibacter* spp., which infects a variety of economically important crops, including potato, maize, and tomato [42]. The peptaibols trichorzianine A1 (Figure 2; 7) and B1 (Figure 2; 8) from *Trichoderma harzianum* could inhibit the spore germination, as well as hyphal elongation, of plant pathogenic fungi [43,44], and there was a synergistic interaction between hydrolytic enzymes and peptaibols [45]. The antiviral properties of the peptaivirins A (Figure 2; 9) and B (Figure 2; 10) belonging to the peptaibol group has also been reported against tobacco mosaic virus infection in tobacco plants [46]. Peptaibols induce plant defense reactions through the salicylate signal pathway, leading to systemic acquired resistance, which is an interesting feature [47,48,49]. The potential of the peptaibols of *Trichoderma* qualifies their exploitation as important plant protectants. There are two peptaibol synthetases (of 18 and 14 modules) in *Trichoderma* genomes. Even though there are more than 700 described peptaibol sequences [50], no genetic studies on their synthesis have been conducted, except in *T. virens* Gv29-8. Using gene disruptions, the 18-residue peptaibol synthetase Tex1 has been shown to be responsible for the production of the trichovirin II-type 18-residue peptaibol, while the 14-module enzyme assembles both the 14-residue and the 11-residue peptaibol in *T. virens* [28,51,52]. 

### 2.3. Pyrones 

**Figure 11:**
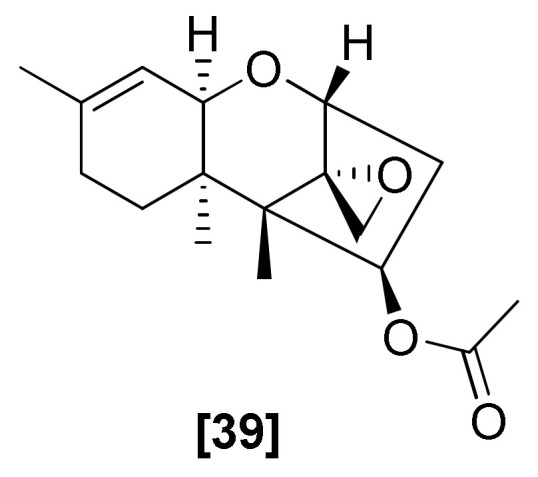
Structure of antifungal trichothecene: [39] trichodermin from *Trichoderma* spp.

The pyrone 6-pentyl-2H-pyran-2-one (6-PP) (Figure 3; 11) is a flavoring agent responsible for the aroma of coconut and has been reported to have antifungal and plant growth-promoting activities [53]. It belongs to the chemically diverse group of low molecular weight metabolites having a high vapor pressure at room temperature and low water solubility, which are classified as volatile organic compounds (VOCs) [54]. Pyrone 6-PP was first discovered in a culture broth of *T. viride* [55], after which it was also reported to be produced by *T. koningii* and *T. harzianum* [56,57]. It caused 31.7% and 69.6% growth reduction in *F. oxysporum* and *R. solani*, respectively, at a concentration of 0.3 mg/ml. A positive antifungal correlation had been investigated between pyrone 6-PP production and the antagonistic ability of *T. harzianum* [58,59]. In stored kiwi fruits, the application of pyrone 6-PP at 0.4 to 4 mg /mL could significantly reduce *B. cinerea* rots on both naturally infected and artificially inoculated fruits [60]. In addition, 6-PP was also found in *T. harzianum* T77 and SQR-T037, which were used for the control of grapevine trunk diseases [61] and *Fusarium* wilt in cucumber in continuously cropped soil [62]. *T. harzianum* was found to produce three bioactive analogues of pyrone 6-PP (Figure 3; 12–15). The analogue (12) was active against *Candida albicans*, *Penicillium* spp., *Cryptococcus neoformans*, and *A. fumigatus* [56,63]. In another study, analogue (12), isolated from *T. harzianum* and *T. longibrachiatum*, exhibited antifungal activity against *Armillaria mellea* [64]. The analogue hydro-derivatives massoilactone (13) and *d*-decanolactone (14) were reported to have activity against *Phytophtora* and *Botrytis* species [65]. Another analogue of pyrone, viridepyronone (15), was produced by a strain of *T. viride* and showed 90% growth inhibition of *S. rolfsii* at a minimum inhibitory concentration (MIC) of 196 mg/ml [66]. Pyrone 6–PP and its analogues are derived from fatty acids, and their biosynthesis in *T. atroviride* IMI206040 has been studied by using [1-^14^C] and [U-^14^C] linoleic acid. It was suggested that the oxidization of linoleic acid to 13-hydroperoxide-diene, followed by 5-hydroxy-2,4-decenioc acid formation and finally esterification, resulted in the formation of pyrones [67].

### 2.4. Butenolides

An antifungal butenolide compound, harzianolide (Figure 4; 16), was isolated from three strains of *T. harzianum* [68,69,70]. The dehydro-derivative (17) of harzianolide (16) was obtained from *T. harzianum.* Another butanolide, T39butenolide (18), was produced by a commercially available *T. harzianum* strain [71]. All of these compounds (16–18) showed antifungal activity against *Gaeumannomyces graminis* var. *tritici* [68,71]. Harzianolide (16) particularly inhibited the growth *G. graminis* var. *tritici* at 200 mg/mL, while T39butenolide (18) inhibited the growth of *G. graminis* at 100 mg/mL. Additionally, harzianolide (16) and T39butenolide (18) caused growth inhibition in *P. ultimum* and *R. solani* [71]. From the fungus *T. longibrachiatum* Rifai aggr, 5-Hydroxyvertinolide (19) was isolated, which was antagonistic to the fungus *Mycena citricolor*, the agent responsible for American leaf spot disease of coffee [72]. In another study, the antifungal effect of a compound of harzianolide (16) and T39butenolide (18) was reported against *P. ultimum*, *R. solani*, and *B. cinerea* [73]. The biosynthesis of these butenolides probably involves two Favorskii rearrangements from a C-14-diepoxide, resulting in the extrusion of the two carbons that form the lactone [74].

### 2.5. Pyridones

Antifungal harzianopyridone (Figure 5; 20) was first isolated from *T. harzianum* in 1989. It contains a pyridine ring system with a 2,3-dimethoxy-4-pyridinol pattern [75]. The racemic form of harzianopyridone (20) showed strong antifungal activity against plant pathogenic fungi, such as *P. ultimum, G. graminis* var. *tritici* [71], *R. solani*, and *B. cinerea* [75]. A laevorotatory form of harzianopyridone (20) isolated from *T. harzianum* exhibited weak antibacterial and antifungal activity and also showed high phytotoxicity in an etiolated wheat coleoptile bioassay analysis. The harzianopyridone (20) was also reported to cause necrosis in corn, bean, and tobacco in a concentration-dependent manner, which suggested that the two harzianopyridone (20) enantiomers may exhibit different activities [74]. In another investigation, harzianopyridone (20) isolated from *T. harzianum* showed activity against *Phytophthora cinnamomi, B. cinerea*, and *Leptosphaeria maculans* [73]. This compound was also reported to inhibit more than 90% of the growth of *R. solani*, *F. oxysporum*, and *S. rolfsii* [76]. The pyridone harzianopyridone (20) was proposed to be biosynthesized from a tetraketide with the possible involvement of aspartic acid [74,75].

### 2.6. Azaphilones

The azaphilones contain a chiral quaternary center and extremely high oxygenated bicyclic core, and hence form a structurally diverse group of SMs. Two azaphilone-type compounds, harziphilone (Figure 6; 21) and fleephilone (Figure 6; 22), were reported to be produced by *T. harzianum*. These were isolated by the bioassay-guided fractionation of the butanol–methanol extract of the fermentation broth of *T. harzianum*. *T. harzianum* was also found to produce another azaphilone, T22azaphilone (23). These compounds exhibited significant antifungal activity against *P. ultimum, G. graminis* var. *tritici*, and *R. solani* [71]. T22azaphilone (23) also exhibited antifungal activity against *B. cinerea, P. cinnamomi*, and *L. maculans* at low doses [73]. Gene deletions and biochemical investigations demonstrated that azaphilones were collaboratively synthesized by two separate clusters containing four core enzymes, two nonreducing PKSs, one highly reducing PKS, and one NRPS-like PKS. This is a meaningful mechanism of fungal SMs, which allows fungi to synthesize more complex compounds and gain new physiological functions [77].

### 2.7. Koninginins

Some species of *Trichoderma* produced a series of SMs, named koninginins A–E (Figure 7; 24–28) and G (29). Koninginins A (24) and B (25) were identified in the culture broth of a strain of *T. koningii* obtained from soil and the root of *Diffenbachia* species [78,79]. Two strains of *T. harzianum* isolated from wheat roots were also reported to produce koninginins A (24) and B (25) in their liquid cultures [68]. The total synthesis of compounds 24 and 25 allowed for the correction of the relative configurations of koninginins A (24a) and B (25a) [80,81]. Later, in 2002, X-ray analysis was used to confirm this stereochemistry [82]. The koninginins C (26) and D (27) were produced by *T. koningii* isolated from soil and fermented on a shredded wheat medium [83,84]. The koninginin E (28) was isolated from liquid cultures of *T. harzianum* and *T. koningii* [85,86] and koninginin G (29) was obtained from *Trichoderma aureoviride* [87]. The total synthesis of koninginin D (27) and E (28) has been performed [81]. Except for koninginin C (26), all other koninginins are bioactive against different plant fungal pathogens. For example, koninginins A, B, D, E, and G (24, 25, 27, 28, 29) exhibit activity against *G. graminis* var. *tritici* [68,85], while koninginin D (27) was reported to have antifungal activity against several plant pathogenic fungi, such as *F. oxysporum, Bipolaris sorokiniana, P. cinnamomi*, and *Pythium middletonii* [84]. In another study, koninginins A, B, and D (24, 25 and 27), obtained from *Trichoderma koningiopsis* YIM PH30002, exhibited antifungal activity against *F. oxysporum*, *Fusarium solani*, and *Alternaria panax* [88]. Koninginins belong to the secondary metabolite group of polyketides. Generally, the polyketide synthases catalyze the polyketide biosynthesis reaction, which is carried out by the repeated attachment of short chain fatty acids, i.e. propionate and acetate, by similar pathways exhibited by fatty acid biosynthesis [89].

### 2.8. Steroids

Stigmasterol (Figure 8; 30) was obtained from *T. harzianum* and *T. koningii* that showed antifungal activities against *R. solani, S. rolfsii, M. phaseolina*, and *F. oxysporum* [76,90]. Two other steroids, ergosterol (31) and 3,5,9-trihydroxyergosta-7,22-dien-6-one (32), isolated from *Trichoderma* sp. YM 311505, exhibited strong antifungal activities against *Pyricularia oryzae*, *C. albicans*, *Aspergillus niger*, and *Alternaria alternata* with an MIC value of 32 µg/mL [91]. 

### 2.9. Anthraquinones

Three anthraquinones, 1,8-dihydroxy-3-methylanthraquinone (Figure 9; 33), 1-hydroxy-3-methylanthraquinone (34), and 6-methyl-1,3,8-trihydroxyanthraquinone (35), were isolated from *T. harzianum* strains that were active against *R. solani*, *S. rolfsii*, *M. phaseolina*, and *F. oxysporum* [76]. Compounds 33 and 34 also showed antifungal activity against *G. graminis* var. *tritici* and *P. ultimum* [71]. It was reported that the low oxidation state of 6-methyl-1,3,8-trihydroxyanthraquinone (35) had the potential to change to a high oxidation state by the host reactive oxygen species that were released in response to attack by microbial pathogens, which means compound 35 may have the ability to increase the efficiency of *Trichoderma* against host resistance to other pathogens [92]. 

### 2.10. Lactones

The antifungal 10-member lactone cremenolide (Figure 10; 36) was isolated from *T. cremeum*. Along with the promotion of tomato seedling growth, this compound (36) also showed antifungal activities against *R. solani*, *B. cinerea*, and *F. oxysporum* [93]. Another lactone, aspinolide C (37), was isolated from *T. arundinaceum* and showed an antibiotic effect against *B. cinerea* and *Fusarium sporotrichioides*. Beside its direct antibiotic effect, compound (37) also played an important role in the induction of plant resistance against phytopathogenic fungi. [94]. Cerinolactone (38) was isolated from culture filtrates of *T. cerinum* [95] and showed strong activity against *Rosellinia necatrix* [96].

### 2.11. Trichothecenes

Trichothecenes are the sesquiterpenoid-derived SMs mainly produced by *Fusarium* and other fungal genera, like *Trichoderma*, *Trichothecium*, and *Stachybotrys* [97,98]. The chemical structure of trichothecenes comprises a trichothecene ring, which contains an olefinic ring at C-9,10, and an epoxide group of C-12 [97]. Trichothecenes inhibit protein synthesis by preventing peptide bond formation at the peptidyl transferase center of the 60S ribosomal subunit [99,100]. Trichodermin (Figure 11; 39) was the most widely studied antifungal compound [99,100]. It was first obtained from *T. brevicompactum* and displayed significant inhibitory activity on *R. solani*, *B. cinerea*, and *Colletotrichum lindemuthianum* (EC_50_ = 25.60 g/mL) [97]. It was also isolated from *T. harzianum* and showed activities against several phytopathogenic fungi, such as *Cochliobolus miyabeanus*, *R. solani*, *C. lindemuthianum*, *F. oxysporum*, *Thanatephorus cucumeris*, *Colletotrichum gloeosporioides*, and *B. cinerea* [101,102]. 

### 2.12. Others

**Figure 12:**
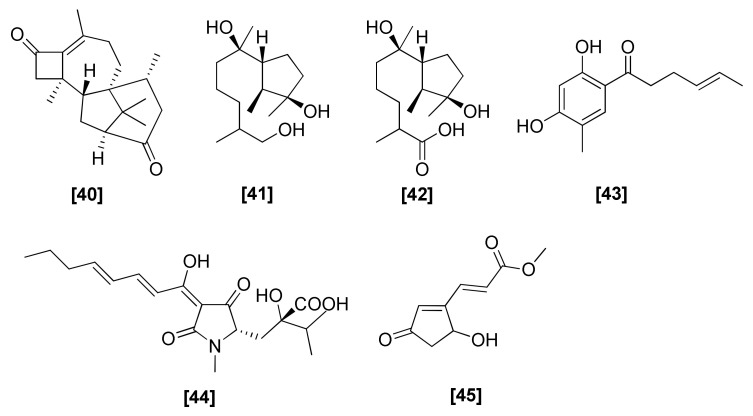
Structures of other antifungal compounds from *Trichoderma* spp.: [40] harziandione, [41] 10,11-dihydrocyclonerotriol, [42] catenioblin C, [43] sohirnone A, [44] harzianic acid, [45] trichodermester A.

Other antifungal compounds belonging to different chemical classes isolated from *Trichoderma* spp. are briefly described here, and their structures are presented in Figure 12. Diterpene harziandione (Figure 12; 40) was isolated from *T. harzianum* [103] and *T. viride* and showed antifungal activity against *S. rolfsii* [104]. Three antifungal compounds, 10,11-dihydrocyclonerotriol (41), catenioblin C (42), and sohirnone A (43), were obtained from *T. longibrachiatum* and have been shown to have antifungal activities against *C. albicans* and *P. oryzae* [105]. Harzianic acid (44), a tetramic acid produced by the *T. harzianum* M10 strain, demonstrated remarkable biological properties, including plant growth promotion and antimicrobial activity against different plant pathogenic fungi, such as *Pythium irregulare*, *Sclerotinia sclerotiorum*, and *R. solani* [106]. The cyclopentenoneacrylic acid derivative trichodermester A (45) was isolated from a marine-derived *T. atroviride* and showed activity against *Phaeosphaerella theae* with an MIC of 125 µg/disc [107]. 

## 3. Antifungal Mechanisms of *Trichoderma* SMs 

The success of *Trichoderma* spp. for their antifungal activities against phytopathogenic fungi could be attributed to the combined action of SMs and hydrolytic enzymes [108]. The inhibition of *B. cinerea* spore germination has been shown to be due to the synergetic effect of gliotoxin (Figure 1; 1) and endochitinase enzymes [109], while *gliP-*deleted mutants of *T. virens*, which are unable to produce gliotoxin, reduced their mycoparasitism against the soybean pathogen *S. sclerotiorum* and oomycete pathogen *P. ultimum* [27]. Similar to other plant beneficial microorganisms, *Trichoderma* fungi release elicitor-like substances which induce a systemic or localized resistance response in plants [5]. 

Various SMs produced by *Trichoderma* spp., such as harzianolides, peptaibols, and certain volatile compounds, are reported to have antifungal potential, as well as acting as a plant growth promoter, resulting in increased plant resistance to pathogen attack. For example, 6-PP (Figure 3; 11), along with reducing the mycelial growth of *F. oxysporum*, *B. cinerea*, and *R. solani*, also promotes plant growth and induces systemic resistance, probably by acting as an auxin-like compound [53]. Recently, it has been shown that tomato plants treated with 6-PP produced significantly more *γ*-aminobutyric acid and acetylcholine, which helps the plants to resist pathogens [110]. The antifungal activities of peptaibols are due to their ability to form ion channels in membranes and inhibit the enzymes responsible for the synthesis of cell walls [111,112,113]. Trichokonin VI (Figure 2; 4), a peptaibol derived from *T. pseudokoningii*, showed antifungal activity by inducing extensive apoptotic programmed cell death [41,114]. In addition, peptaibols also trigger plant defense responses. The Dtex1-deleted mutants of *T. virens*, which were unable to produce 18-residue peptaibol, failed to trigger systemic resistance responses in cucumber [28]. Meanwhile, the application of the 20-residue peptaibol alamethicin F30 (Figure 2; 3) from *T. viride* induced jasmonic acid- and salicylic acid-mediated resistance in lima bean [47]. 

Another mechanism of SMs for controlling phytopathogenic fungus is their role in the competition for nutrients. The fast-growing ability of *Trichoderma* spp. makes them potential competitors for nutrients and space. *Trichoderma* spp. make iron unavailable for the competing microorganisms by releasing siderophores, which scavenge iron from the environment. Iron competition has been shown to play an important role in the antagonistic activity of *T. asperellum* against *F. oxysporum* [115]. The coiling ability of *Trichoderma* around thehyphae of the prey fungus increases its mycoparasitism activity [116]. It is reported that the anthraquinone SMs, emodin and pachybasin, derived from *T. harzianum*, play a role in the self-regulation of coiling in *T. harzianum* [117]. The addition of these compounds increased the number of coils of the mycoparasite around *R. solani* hyphae, and this effect seems to be due to a stimulation of cAMP synthesis. Some SMs interact with the toxins of pathogenic fungi and inhibit their growth. For example, 6-PP (Figure 3; 11) secreted by *T. harzianum* degrades fusaric acid and mycotoxins and inhibits *Fusarium moniliforme* mycelial growth [118].

## 4. Approaches for the Analysis of SMs in *Trichoderma* spp.

**Figure 13:**
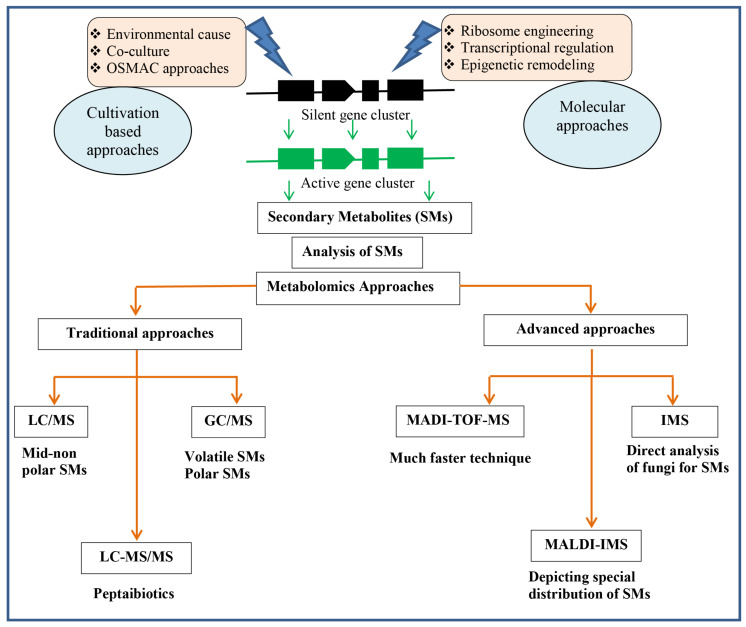
Schematic presentation of approaches for the analysis of SMs in *Trichoderma* spp.

For SMs, there is not a one-to-one relationship between a metabolite and a gene. The secondary metabolome, however, in many cases is a result of many genes and their enzymes [119]. The fungal sequencing of fungal genomes disclosed the fact that gene clusters associated with SMs exceed the number of SMs from a given fungus and several gene clusters from the prediction remain silent [120]. Different molecular, as well as cultivation-based, approaches involved in the regulation of these silent gene clusters can be utilized for their activation [121,122]. Metabolomics, along with the efforts for the activation of silent gene clusters, can contribute to the development and identification of new SMs (Figure 13). Metabolomics includes the untargeted, as well as targeted, approaches for determining the identity of all low molecular weight SMs of an organism. Untargeted approaches are the methods and techniques for the searching of all known and unknown detectable compounds, while targeted approaches are for the identification of already known compounds. Different chromatographic techniques, such as gas and liquid chromatography, along with mass spectrometry, are useful for the analysis of metabolites in complex samples. These techniques are helpful to detect a large number of metabolites. The applications of liquid chromatography mass spectrometry (LC-MS) allows for the detection of mid- to nonpolar metabolites, while gas chromatography mass spectrometry (GC-MS) is suitable for the study of both volatile and polar small substances [123]. Liquid chromatography, when combined with tandem mass spectrometry (LC-MS/MS), is useful for peptaibiotic detection in the samples extracted from fungal cultures, whereby the specific amino acid, Aib, for peptaibiotics can be indicated by mass differences of D m/z 85 [30]. The known structures of peptaibiotics can be obtained by comparing the amino acid sequences obtained from LC-MS/MS analysis with their respective databases, such as the “Comprehensive Peptaibiotics Database” [124]. The matrix-assisted laser desorption/ionization time of flight mass spectrometry (MALDI-TOF-MS) is an advanced approach, which is much faster and more effective than the traditional bioactive screening techniques, to discover new bioactive SMs in fungus. This technique was used for the detection of peptaibol production profiles from 28 different *Trichoderma* species [125]. Imaging mass spectrometry (IMS) is another advancement that allows the direct analysis of fungi for SMs. In association with MALDI and coupled to a mass spectrometer, IMS produces images depicting the spatial distribution of natural products [126,127,128,129]. MALDI-IMS has been used for the metabolic analysis of living bacterial communities and interkingdom interactions between fungi and bacteria directly from their cultures [130,131,132]. Recently, MALDI-IMS was used to visualize the SMs in the mycoparasitic interaction of *R. solani* and *T. atroviride* [133]. Little, or even no, sample preparation requirements make the MALDI techniques well suited to the analysis of co-cultivations [134].

## 5. Biosynthesis Pathway and Factors Affecting the Regulation of SMs in *Trichoderma* spp.

**Figure 14:**
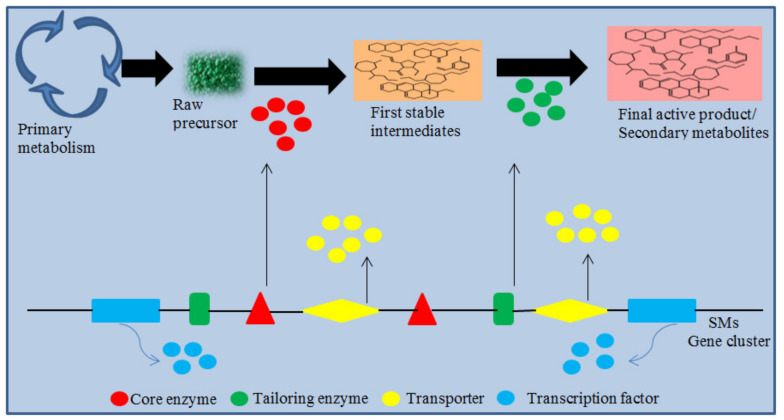
Proposed biosynthetic scheme and the regulation factors of SMs in *Trichoderma* spp.

SMs are usually synthesized from a few precursors produced by primary metabolism, which act as raw material for the production of SMs. These precursors are then transformed to first stable intermediates through the action of different core enzymes. Based on the core enzyme involved in the biosynthesis of intermediates, they can be divided into different groups, such as dimethylallyl tryptophan synthases, polyketide synthases (PKSs), terpene cyclases, non-ribosomal peptide synthetases (NRPSs), and hybrid PKS-NRPS enzymes, and are involved in the production of indole alkaloids, polyketides, terpenes, non-ribosomal peptides, and PKS-NRPS hybrids, respectively [135]. The further modification of the first stable intermediates is generally accomplished by decorating or tailoring enzymes, resulting in the formation of a final active product or compound [135]. In addition to these core enzymes, the gene cluster of SMs may also contain other genes that encode transcription factors for the regulation of gene expression involved in biosynthesis and transporters that contribute to self-protection or SM efflux [135,136,137,138]. The evolutionary force responsible for the maintenance and formation of SM genes in physical clustering is unclear [139]. However, the physically linked genes in a cluster exhibit the ability of better co-regulation, which allows a strong coordinated connection among the enzymes involved in the same biosynthesis pathway [140,141,142]. Here, a brief introduction on the SM biosynthetic scheme and their regulation factors in *Trichoderma* fungi is presented (Figure 14).

Recent studies related to regulatory factors and the influence of environmental conditions on fungal SMs enhanced our understanding on the tightly regulated cellular process of SMs. Like other fungi, in *Trichoderma* spp., different factors, such as pH signaling, velvet-complex proteins, and interactions with other organisms, are responsible for the expression of genes related to SMs [143,144,145,146,147,148,149]. The transcriptomic responses of *T. virens*, *T. reesei*, and *T. atroviride* to the presence of *R. solani* were evaluated, and two PKSs were found among the genes induced in *T. reesei*–*R. solani* and *T. atroviride*–*R. solani* interactions, whereas all the genes in the biosynthesis cluster of gliotoxin were up-regulated [143]. An up-regulation of the lipoxygenase gene, that is involved in the biosynthesis of 6-PP, was also noticed in *T. atroviride* [150]. In another study, the co-culture of *T. arundinaceum* and *B. cinerea* revealed an increase in the expression of *tri* biosynthetic genes [147]. However, in the interaction zone between *T. arundinaceum* and *B. cinerea*, a secondary metabolite of *B. cinerea*, which is also a virulence factor of *B. cinerea*, reduced *tri* gene expression and harzianum A production in *T. arundinaceum* [147]. 

It was reported that the presence of the *Fusarium* mycotoxin fusaric acid resulted in the suppression of 6-PP (Figure 3; 11) production and the induction of sporulation-associated metabolite i.e., 1-octen-3-ol, production [151]. In return, certain *Trichoderma* strains, due to the secretion of 6-PP, are capable of inhibiting fatty acid production by *F. moniliforme* and degrading fatty acids [118]. In *Trichoderma* genomes, gene clusters related to the production of SMs harbor specific transcription factors. In addition to these regulators present in gene clusters, several other key players also take part in the regulation of SM biosynthesis, such as PacC, a pH regulator which influences different fungal genes in response to environmental pH [149,152]. The PacC orthologue of *T. virens* controls the iron transport and biosynthesis of SMs. In DpacC mutants of *T. virens*, the gene expression was altered for cytochrome P450, NRPS Tex15, and siderophore-related biosynthesis enzymes [153]. Moreover, biocontrol activity was reduced in *T. virens* DpacC mutants, which may be because of their inability to adapt to alkaline pH.

The production of SMs is also under the regulation of the heterotrimeric velvet complex. This complex consists of two velvet proteins, VelA and VelB, and methyltransferase LaeA [154]. The *velA* orthologue *vel1* governs the regulation of gene clusters related to the production of SMs. The disruption of the *vel1* gene stopped the biosynthesis of gliotoxin and silenced several SM-related genes that encode for one cytochrome P450 monooxygenase, two PKSs, NRPSs, and one *O*-methyl transferase [148]. A similar role was noticed for the *T. reesei* LaeA orthologue that influenced the expression of lignocellulose-degrading enzymes [146,155]. The *T. atroviride* removal of *lae1* resulted in abolishing the antifungal activity of *T. atroviride*. This correlated with a significantly reduced expression in 6-PP-related lipoxygenase genes and PKS-encoding genes. The influence of *lae1* on the production of 6-PP was also corroborated when, in antagonism experiments, the enhanced production of 6-PP was noticed in *lae1* over-expressing strains [146]. The biosynthesis of 6-PP in *T. harzianum* is also associated with Thctf1. The deletion of the transcription factor Thctf1 altered the antimicrobial activity of *T. harzianum* and abolished the production of two SMs derived from 6-PP [156].

The transfer and sensing of environmental cues affecting the regulation of fungal SMs was achieved by membrane bound receptors, such as G protein-coupled receptors (GPCRs), and their associated intracellular signaling pathways. The *T. atroviride* biosynthesis of SMs was governed by G protein signaling and the associated cAMP pathway [157,158,159]. The decrease in 6-PP and increase in peptaibol production was reported with the deletion of *tga1*, which encodes an adenylyl cyclase-inhibiting Ga subunit of *T. atroviride* [158]. The biosynthesis of peptaibol was further dependent on two regulators, BLR1 and BLR2, under certain conditions [160].

## 6. Conclusions

Fungi, being a most diverse group of phytopathogens, exert a huge impact on agriculture. High genetic flexibility and broad-spectrum lifecycles allow the pathogenic fungi to develop fungicide resistance and invade new hosts. Therefore, new management strategies are needed for fighting against pathogenic fungi. The utilization of SMs from *Trichoderma* spp. has been used in plant protection as an environmentally friendly and efficient management tool against a variety of phytopathogens. This review presented the fungicidal SMs from *Trichoderma* spp. against phytopathogenic fungi. Some aspects of the structural overview of SMs and their biosynthesis were reviewed. Brief information on the biosynthesis pathway, action mechanism, different approaches for the analysis of SMs, and factors affecting the regulation of SMs in *Trichoderma* was also discussed.
